# Prevalence of Toxoplasmosis in Sheep and Goats in Pakistan: A Systematic Review and Meta-Analysis

**DOI:** 10.3390/pathogens11111331

**Published:** 2022-11-11

**Authors:** Tanzila Mumtaz, Usman Ayub Awan, Aqsa Mushtaq, Muhmmad Sohail Afzal, Tahir Mahmood, Samia Wasif, Abid Ali, Kiran Ajmal, Teroj Mohamed, Ali Muhammad, Hua Liu, Haroon Ahmed, Jianping Cao

**Affiliations:** 1 Department of Biosciences, COMSATS University Islamabad (CUI), Park Road, Chakh Shahzad, Islamabad 22620, Pakistan; 2 Department of Medical Laboratory Technology, The University of Haripur, Haripur 31261, Khyber Pakhtunkhwa, Pakistan; 3 Department of Life Sciences, School of Science, University of Management & Technology (UMT), Lahore 22209, Punjab, Pakistan; 4 Department of Industrial and Systems Engineering, College of Computing and Mathematics, King Fahd University of Petroleum and Minerals, Dhahran 31261, Saudi Arabia; 5 Interdisciplinary Research Center for Smart Mobility & Logistics, King Fahd University of Petroleum and Minerals, Dhahran 31261, Saudi Arabia; 6 Department of Humanities, COMSATS University Islamabad (CUI), Islamabad 45550, Pakistan; 7 Department of Zoology, Abdul Wali Khan University Mardan, Mardan 23200, Khyber Pakhtunkhwa, Pakistan; 8 Dental Basic Sciences Department, College of Dentistry, University of Duhok, AJ Duhok 1006, Iraq; 9 Department of Zoology, University of Poonch (UOP), Rawlakot 12350, Azad Jammu and Kashmir, Pakistan; 10 National Institute of Parasitic Diseases, Chinese Center for Disease Control and Prevention (Chinese Center for Tropical Diseases Research), Key Laboratory of Parasite and Vector Biology, National Health Commission of the People’s Republic of China, World Health Organization Collaborating Center for Tropical Diseases, Shanghai 200025, China; 11 The School of Global Health, Chinese Center for Tropical Diseases Research, Shanghai Jiao Tong University, Shanghai 200025, China

**Keywords:** toxoplasmosis, *Toxoplasma gondii*, prevalence, sheep, goat, Pakistan

## Abstract

Toxoplasmosis, a parasitic disease caused by *Toxoplasma gondii*, results in congenital disorders and miscarriages among livestock and humans worldwide. This systematic review and meta-analysis were conducted to determine the prevalence of *T. gondii* infection in sheep and goats in Pakistan from 2000 to 2020. We searched the PubMed, Scopus, EMBASE, and Google Scholar databases and selected 17 publications that fulfilled our inclusion criteria. Eight studies were conducted in Southern Punjab, six in Khyber Pakhtunkhwa, two in Northern Punjab, and one in Central Punjab. The diagnostic tests used in the included articles to confirm toxoplasmosis were the latex agglutination test in 56% of the studies, the enzyme-linked immunosorbent assay in 38%, and the indirect hemagglutination assay in 6%. The infection rates were substantially higher among sheep > 1 year of age (37%) than among sheep ≤ 1 year old (19%). Statistically significant differences in infection rates were found between male and female sheep and goats. The overall infection rate by age was also significant among sheep and goats. Sex and age variability between sheep investigations were significant, and sex heterogeneity and age homogeneity were significant among goats. Hence, robust infection control protocols should be implemented to prevent infection in animals and humans.

## 1. Introduction

Toxoplasmosis is a zoonotic disease caused by *Toxoplasma gondii (T. gondii),* an obligate intracellular protozoan parasite. Toxoplasmosis affects domestic animals (including goats and sheep) and humans and requires combined approaches across disciplinary boundaries [1]. This disease is a significant public health issue for humans, animal agriculture, and livestock [2,3].

The world’s most significant livestock, sheep and goats, are consumed, especially in developing nations [4]. Considering this, toxoplasmosis surveillance and eradication need extensive epidemiological surveys. According to the previous literature, a wide range of serological studies has been undertaken to understand the epidemiology of toxoplasmosis in different regions, including South and North America [5,6,7], Europe [8,9,10], Africa [11,12,13], and Asia [14,15]. However, in Asian countries, such as Bangladesh [16,17,18,19,20], India [21,22,23,24,25], China [26,27,28,29,30,31,32,33], Iran [34,35,36,37], Iraq [38,39,40], Afghanistan [41], and Sri Lanka [42], toxoplasmosis remains a major risk factor for the public health of humans and for livestock.

Pakistan is Asia’s third largest breeding country, with a population of 78.2 million goats and 31.2 million sheep [43]. Pakistan’s livestock sector has become the most significant contributor to agriculture; it contributed approximately 60.6% to Pakistan’s agriculture sector and 11.7% to its gross domestic product (GDP) in 2019–2020, and its goats and sheep have had a significant economic impact on the country. In the fiscal year 2019–2020, Pakistan added approximately 1 million, 0.47 million, 0.75 million, and 0.29 million tons of milk, wool, meat, and hair, respectively, and 59.5 million pounds of small ruminant skin to its total GDP [44].

Small ruminants, such as sheep and goats, are particularly vulnerable to *T. gondii*, resulting in various problems in these animals [45]. Toxoplasmosis causes deficits in health and production through neonatal deaths, stillbirths, and abortions [46], damaging the reproductive system and, thus, negatively affecting the cost-effectiveness of goats and sheep [47]. Toxoplasmosis was unknown in sheep and goats until the first case was reported by Feldman and Hartley [2,48]. The primary source of infection was determined to be the oocysts shed by cats in their feces, indicating that wild and domestic cats are the definitive hosts [2,49].

Humans contract toxoplasmosis by consuming undercooked/raw meat or food contaminated with oocysts excreted by cats [50]. Approximately 33% of the global population has been identified as infected with toxoplasmosis [51], and drinking unpasteurized and unboiled sheep and goat milk has been identified as the cause of human toxoplasmosis [48,52,53,54]. Food animals, goats, and sheep (small ruminants) are the most highly afflicted species among mammals with infection of *T. gondii* [55], and they are potential pathways for human disease transmission [56].

Toxoplasmosis has a detrimental effect on the national economy and poses risks to the health of humans. In Pakistan, detailed data are limited despite the high frequency of the disease; therefore, a systematic review of the current research literature and meta-analysis was performed to estimate the prevalence of *T. gondii* infection in goats and sheep and its relationship with various risk factors to identify gaps in the research literature and highlight future research opportunities to improve our knowledge and control of toxoplasmosis in the sheep and goats of Pakistan

## 2. Results

Our search for relevant research literature published over two decades (from 2000 to 2020) yielded 17 articles for inclusion in this study (Table 1). All of the included articles were cross-sectional studies that examined the frequency of toxoplasmosis in various regions of Pakistan. The enzyme-linked immunosorbent assay (ELISA) and latex agglutination test (LAT) diagnostic tests were used in 15 of the 17 studies, and the 2 remaining studies used the indirect hemagglutination assay (IHA) diagnostic test.

**Figure 1:**
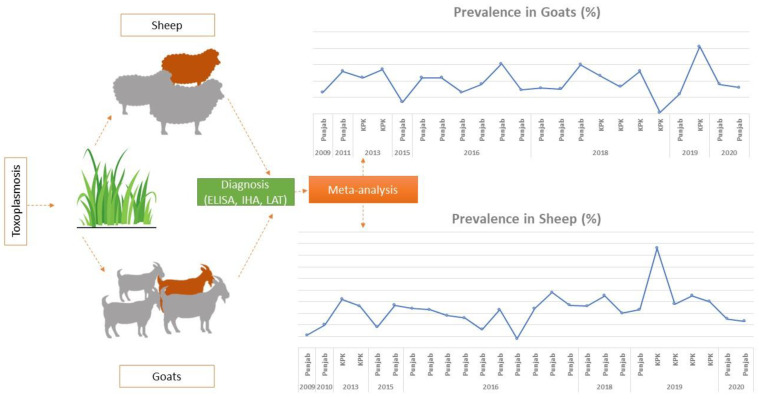
The overall study design and graphs of the prevalence of toxoplasmosis among sheep and goats.

Our study consisted of 3630 sheep, of which 1124 were positive for toxoplasmosis, and a total of 3128 goats, of which 1112 were infected. Hence, the overall prevalence of toxoplasmosis was 35.5% among the goats and 30.9% among the sheep (Figure 1). The incidence of T. gondii infection among the sheep and goats varied geographically; the highest prevalence of infection among the sheep was observed in Charsadda, Khyber Pakhtoon Khwa (KPK), followed by various districts in Punjab province, including 60% in Jalalpur, 48% in Sargodha, and 45% in Shujbad. Infection among the goats was most prevalent in the district of Charsadda, followed by districts in Punjab province, such as 61% in Sargodha, 60% in Jalalpur, and 53% in Mohmand Agency, KPK (cf. Table 2)

Twelve of the fourteen included articles on sheep investigated the prevalence of toxoplasmosis by sex (Table 3). In some of the studies, the statistical analyses revealed a strong correlation between gender and toxoplasmosis, and in a few other studies, no significant difference was found between them. However, a small difference in infection rates by gender was found between male (29%) and female (31%) sheep, compared to a larger difference between male (24%) and female (47%) goats.

Significant differences in infection rates among sheep were reported in studies from different geographical areas, including Multan (females: 65%; males: 25%), Rahim Yar Khan (females: 17%; males: 4.5%), and Dera Ismail Khan, Multan, and Khanewal (females: 18%; males: 30%). Similarly, only a few of the included studies reported a significant difference in the rates of infection among male and female goats, such as Mohmand Agency (females: 69%; males: 38%); Multan (females: 55%; males: 25%); Bhalwal, Kotmomin, Sahiwal, Shahpur, Silanwali, and Sargodha (females: 50%; males: 23%); and Rahim Yar Khan (females: 37%; males: 62%) (cf. Table 3)

Two of the ten studies on sheep were not included in the meta-analysis due to the variation in the data of their age groups. Therefore, eight studies were included, and their findings proved that the infection rates increased with age. In contrast, two of the seven articles on goats were not included in the meta-analysis due to the variation of the data on their age groups; thus, five studies were included (Table 4). Four of the five studies conducted on goats reported a strong positive correlation with age. The findings indicated that the infection rates were significantly greater (37%) among sheep older than 1 year compared to the 19% infection rate among sheep-bearing age (younger than 1 year).

**Figure 2:**
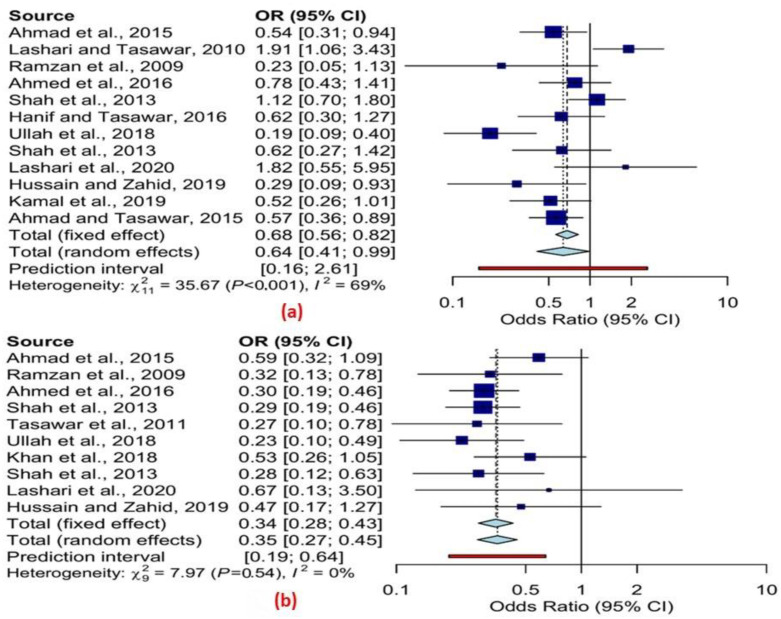
Forest schematic graph for overall prevalence odds ratio and 95 % CI of *T. gondii* infection by gender (**a**) sheep (**b**) goats (random effects) [47,54,55,57,62,64,65,67,68,69,70,71,72].

The results of the fixed-effects model showed a statistically significant difference in infection rates between male and female sheep (i.e., odds ratio (OR) 0.67, 95% confidence interval (CI) 0.56–0.82) and goats (OR 0.34, 95% CI 0.28–0.43) (cf. Table 5 and Table 6). Similar findings were observed in the random effects model for both sheep (OR 0.64, 95% CI 0.41–0.99) and goats (OR 0.35, 95% CI 0.27–0.45) (cf. Table 5 and Table 6). Extensive variation among the different studies in the prevalence estimates of infection among the sheep was observed in the analysis by sex, and the Q statistic was 35.67, *p* < 0.001, and *I*^2^ = 69% (Figure 2a). The Q statistic of 7.97, *p* = 0.54, and *I*^2^ = 0% in Figure 2b revealed no significant variation in the prevalence estimates of infection among goats in the different studies.

**Table 5:** Overall prevalence of *T. gondii* infection among sheep by sex.

Author(s)	Male	Female	OR	95% CI	%W (Fixed)	%W (Random)
	Infected/Total	Infected/Total				
Ahmad et al. [66]	20/156	55/257	0.5401	[0.3097; 0.9418]	13.4	9.8
Lashari and Tasawar [58]	19/63	84/455	1.9072	[1.0595; 3.4332]	5.3	9.6
Ramzan et al. [57]	2/44	8/46	0.2262	[0.0452; 1.1321]	2.8	4.2
Ahmed et al. [62]	16/72	107/398	0.777	[0.4272; 1.4132]	9.5	9.5
Shah et al. [61]	55/120	73/170	1.1243	[0.7025; 1.7995]	12.1	10.3
Hanif and Tasawar [60]	10/51	127/449	0.6184	[0.3007; 1.2719]	7.7	8.7
Ullah et al. [47]	16/63	40/62	0.1872	[0.0867; 0.4043]	11.2	8.4
Shah et al. [61]	16/52	20/48	0.6222	[0.2734; 1.4159]	5.3	8
Lashari et al. [68]	5/15	19/88	1.8158	[0.5538; 5.9541]	1.4	5.9
Hussain and Zahid [66]	25/33	64/70	0.293	[0.0923; 0.9299]	3.7	6.1
Kamal et al. [67]	26/78	32/65	0.5156	[0.2621; 1.0145]	8.6	9
Ahmad and Tasawar [55]	52/169	73/166	0.5662	[0.3618; 0.8862]	18.9	10.5
					z|t	*p*-value
Total (fixed effects)	262/916	702/2274	0.6794	[0.5626; 0.8205]	−4.02	< 0.0001
Total (random effects)	262/916	702/2274	0.6367	[0.4114; 0.9853]	−2.28	0.0439

OR, odds ratio; CI, confidence interval.

**Table 6:** Overall prevalence of *T. gondii* infection in goats by sex.

Author(s)	Males	Females	OR	95% CI	%W (Fixed)	%W (Random)
	Infected/Total	Infected/Total				
Ahmad et al. [59]	16/153	44/266	0.5893	[0.3200; 1.0850]	9.6	12.8
Ramzan et al. [57]	10/62	18/48	0.3205	[0.1311; 0.7837]	5.7	7.4
Ahmed et al. [62]	35/150	192/380	0.298	[0.1942; 0.4574]	27.8	18.9
Shah et al. [61]	39/150	109/200	0.2933	[0.1854; 0.4641]	23.1	17.7
Tasawar et al. [54]	5/20	99/180	0.2727	[0.0951; 0.7824]	5	5.7
Ullah et al. [47]	15/63	36/62	0.2257	[0.1047; 0.4867]	9.2	9.3
Khan et al. [64]	18/56	44/93	0.5275	[0.2638; 1.0548]	7.5	10.8
Shah et al. [61]	20/52	36/52	0.2778	[0.1234; 0.6255]	7.4	8.6
Lashari et al. [68]	2/8	31/93	0.6667	[0.1271; 3.4970]	1.2	2.5
Hussain and Zahid [66]	21/29	78/92	0.4712	[0.1745; 1.2722]	3.4	6.3
					z|t	*p*-value
Total (fixed effects)	181/743	687/1466	0.3445	[0.2780; 0.4269]	−9.74	<0.0001
Total (random effects)	181/743	687/1466	0.3510	[0.2746; 0.4488]	−9.64	<0.0001

OR, odds ratio; CI, confidence interval.

**Figure 3:**
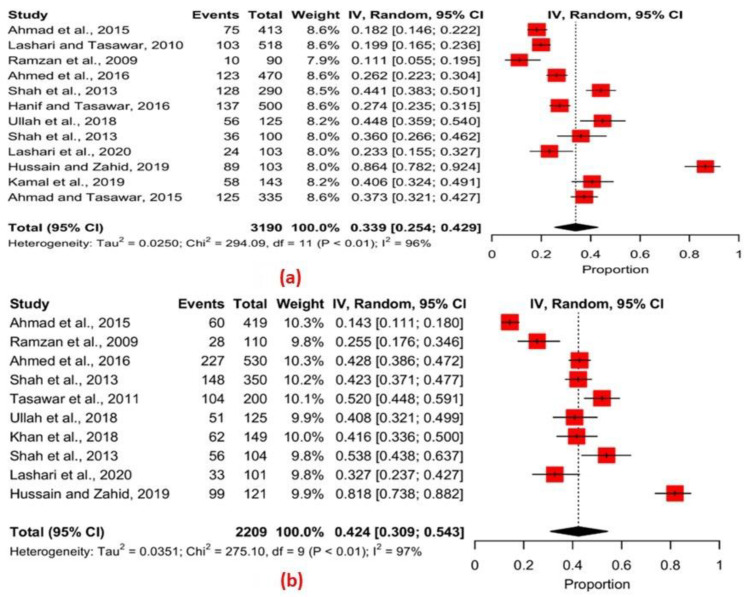
Forest plots showing the proportion of *T. gondii* infection by gender (**a**) sheep (**b**) goats (random effects) [47,54,57,62,64,65,67,68,69,70,71,72].

Using the data analyzed by sex, a forest plot was drawn based on the proportion of infected cases among the male and female sheep (Figure 3a). The Q statistic of 294.09, *p* < 0.01, and *I*^2^ = 96% show significant variation among the different studies in the prevalence estimates of infection among the sheep. The Q statistic of 275.10, *p* < 0.01, and *I*^2^ = 97% (cf. Figure 3b) show significant variation among the different studies in the prevalence estimates of infection among the male and female goats.

**Figure 4:**
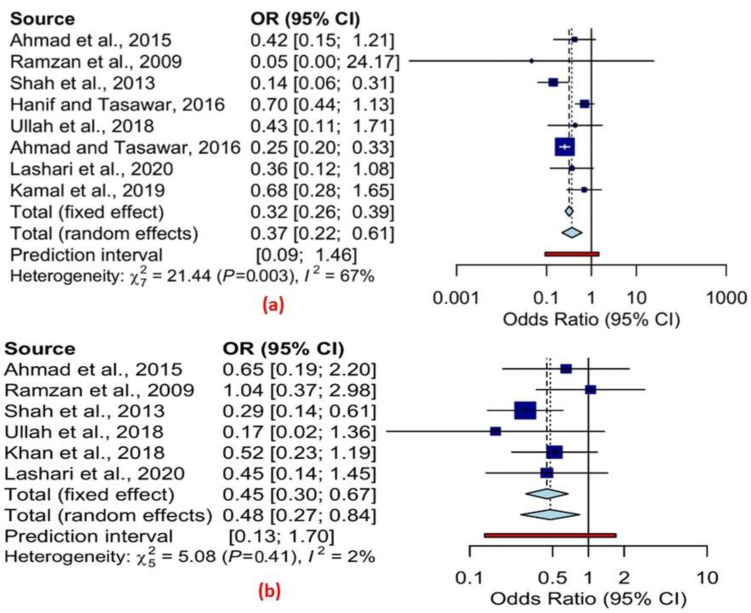
Forest schematic graph for overall prevalence odds ratio and 95 % CI of T. gondii infection by age group (**a**) Sheep (**b**) Goats [47,57,64,65,66,67,68,70,72].

We divided the animals into two groups, i.e., group 1 (≤1 year of age) and group 2 (>1 year of age) to analyze the data by age. The fixed-effects model showed a significant difference in the infection rates between the two age groups of the sheep (OR 0.32, 95% CI 0.26–0.39) and goats (OR 0.44, 95% CI 0.30–0.67) (cf. Table 7 and Table 8). Similar findings using a random effects model were also observed in sheep (OR 0.37, 95% CI 0.22–0.61) and goats (OR 0.48, 95% CI 0.30–0.84) (cf. Table 7 and Table 8). Moreover, extensive variation among the different studies in the analysis by age was observed for the prevalence estimates of infection among sheep, based on the Q statistic of 21.44, *p* = 0.003, and *I*^2^ = 67% in Figure 4a. The Q statistic of 5.08, *p* = 0.40, and *I*^2^ = 2% in Figure 4b showed no significant variation among the different studies in the prevalence estimates of infection among goats.

**Figure 5:**
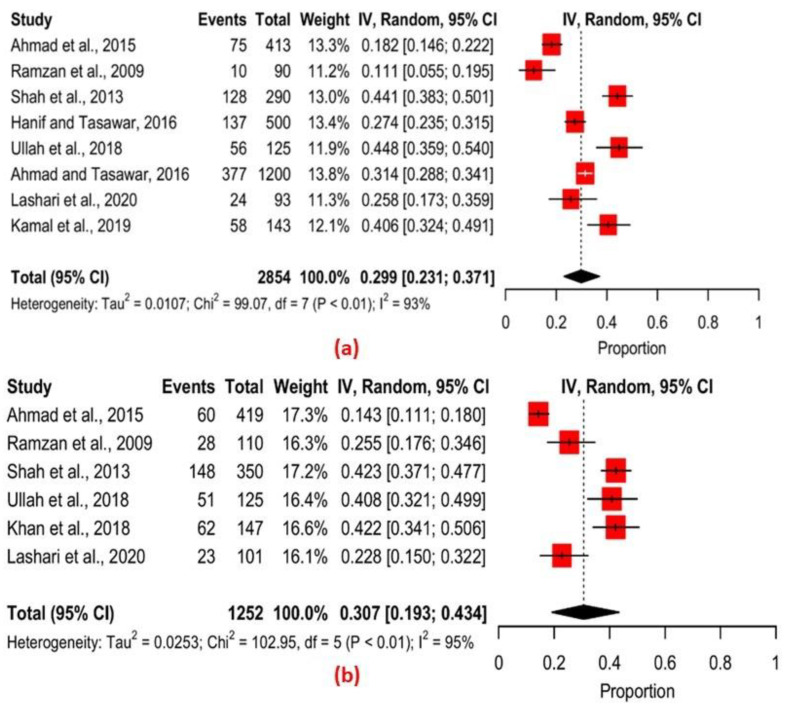
Forest plots showing the overall prevalence of *T. gondii* infection by age group; (**a**) sheep (**b**) goats [47,57,64,65,66,67,68,70,72].

Further analysis by the age of the proportion of infected cases was conducted. The Q statistic of 99.07, *p* < 0.01, and *I*^2^ = 93% in Figure 5a revealed significant variation among the different studies in the prevalence estimates of infection among sheep. The Q statistic of 102.95, *p* < 0.01, and *I*^2^ = 95% in Figure 5b also showed significant variation in the prevalence estimates of infection among the different studies of goats.

## 3. Discussion

Over several decades, Pakistan’s livestock industry has become a vital subsector of the country’s agricultural sector. Goats and sheep are used for various purposes, including producing meat, milk, and other dairy products and breeding. Goat and sheep populations have increased substantially during the last 3 years, as have meat and milk production from sheep and goats. Goats and sheep are most frequently infected among livestock with toxoplasmosis [57], and the primary route of *T. gondii* infection is cat feces; however, it may be transported through the consumption of tissue cysts in raw foods and undercooked foods [73]. The prevalence of toxoplasmosis among livestock varies significantly worldwide, ranging from 0 to 100% in different nations [74,75], depending on the country’s traditions, customs, lifestyle, meteorological conditions, farming practices, and the age of the animal [76]. Prevalence rates are related to the oocyst excretion of cats and the infection of animals and humans after sporulation [3].

After searching four databases, 17 articles, including 3630 sheep with a positive case count of 1124 and 3128 goats with a positive case count of 1112, were selected for the analysis. The data showed that the overall prevalence of infection with toxoplasmosis was 35% among goats and 30.9% among sheep. The peak incidence of *T. gondii* was 86.4%, which was reported in KPK (Shabqadar, Tangi, and Charsadda), and the lowest prevalence rate was 1.47% in the Bannu district [66,77]. The studies reported a significant association between toxoplasmosis infection and the ages of goats and sheep. The higher disease prevalence in animals older than 1 year may be caused by longer exposure throughout their lives (Table 7 and Table 8) [47,57,60,61,63,67,68].

In countries other than Pakistan, the frequency of toxoplasmosis infection in goats and sheep varies. Prevalences of 33.62% for sheep and 36.41% for goats were observed in Iran [78], and a more recent study reported a prevalence of 14.4% among sheep and 8.8% among goats [35]. Seroprevalence, which was reported to be 52.6% among sheep and 24% among goats in Nazareth, Ethiopia, was confirmed by the modified agglutination test, and seroprevalence of 56% among sheep and 25.9% among goats were verified by the ELISA test in the same region [79]. The seropositivity of *T. gondii* in China was reported in 9.84% of sheep and 10.73% of goats [27], and another study reported it in 9.9% of goats [80].

The findings of the fixed-effects model indicated a significant difference in the infection rates between male and female sheep (OR 0.67, 95% CI 0.56–0.82) and goats (OR 0.34% CI 0.28–0.43) (cf. Table 5 and Table 6). The results of the present study are consistent with those of previous studies [54,61,81,82,83], although the results of two studies [69,84] that reported higher seropositivity in males than females were inconsistent with our findings. Females are more susceptible to protozoan parasites than males are. The stress of lactation and childbirth causes immunological suppression in female sheep and goats, predisposing them to toxoplasmosis [70,85,86,87].

The included articles reported that infection rates were significantly higher among sheep more than 1 year of age (37%) compared to sheep less than 1 year (19%) of age (cf. Table 4). The risk factors for toxoplasmosis were more prevalent among older sheep than younger sheep, implying that animals have a greater probability of infection as they age, which is consistent with the study by Shah et al. [61]. The higher susceptibility of older animals than younger animals to infection is thought to be related to their longer exposure to risk factors for infection, and the observations of sheep and goats in other studies are consistent with those of our study [54,58]. However, our findings contradict those of Ramzan et al. [57]. Seroprevalence increases with age due to an increased risk of environmental contamination [88]. This increase could be related to the inability of animals 1 year and younger to retain adequate passive immunity transferred from their mothers. Therefore, the age of the animal is regarded as an essential risk factor for toxoplasmosis infection in animals [89].

In the current study, the overall prevalence of toxoplasmosis in Pakistan was 35.5% among goats and 30.9% among sheep. A higher *T. gondii* seropositivity was observed in goats than in sheep in Northern Punjab [59]. Similarly, the results of other studies indicated that goats are more vulnerable to toxoplasmosis than sheep, owing to increased mobility and migration [57,90], which may have increased their probability of coming into contact with contaminated sources. These correlations may be explained by the constant grazing of the many sheep flocks in the included articles, whereas the goat herds were confined to houses. As a result, the potential for contacting contaminated food and grasslands was higher among the sheep flocks throughout the grazing season. In Pakistan and other countries worldwide, there is significant variation in the occurrence of toxoplasmosis. The findings of our study indicate that toxoplasmosis is widespread in goats and sheep in Sargodha, Sahiwal, Bhalwal, Silanwali, and Shaahpur. Similar results have been reported in Pakistan’s southern areas [54,57,58], KPK [61] and Iran, India, and China [71,72,91]. This substantial variation in the seroprevalence of toxoplasmosis between regions is attributed to differences in temperature, sanitary conditions, farming techniques, sample size, and diagnostic techniques [72].

There are some limitations in this systematic review and meta-analysis. In the different studies, the sex of the animals was not examined equally; hence, uneven samples from each sex were included in the analysis, which may have biased the results and conclusions of the study. Finally, age, which is a critical factor, was not investigated in all of the included articles.

## 4. Materials and Methods

### 4.1. Data Search Strategy

Our study was a detailed investigation of the prevalence of *T. gondii* infection among goats and sheep in Pakistan. We collected data from searches of four databases, including Google Scholar, Scopus, EMBASE, and PubMed, and analyzed relevant findings identified in the articles. Keywords, including “toxoplasmosis”, “*T. gondii*”, “prevalence”, “sheep”, “goat”, and “Pakistan” were searched alone or in combination in the four databases.

### 4.2. Data Collection

Research articles written in English were selected for review. All the study’s authors conducted the systematic review and meta-analysis of the included articles, gathered research reports, and defined the study’s inclusion criteria.

### 4.3. Inclusion and Exclusion Criteria

**Figure 6:**
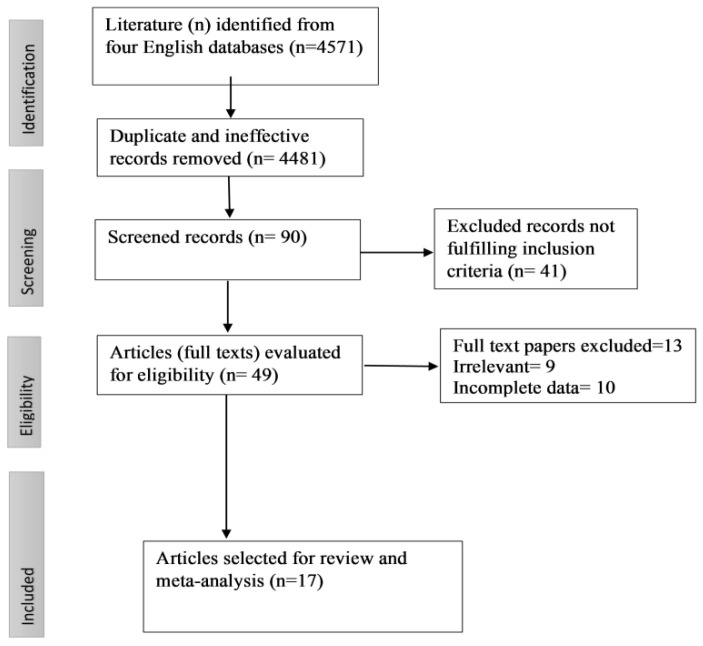
PRISMA flowchart of the selection of studies.

The exclusion and inclusion criteria were evaluated, and the articles for inclusion were selected accordingly. Studies conducted in Pakistan that investigated the prevalence of toxoplasmosis in the country’s sheep and goats were screened and evaluated for inclusion in the systematic review and meta-analysis. Irrelevant data, incomplete information, duplicate articles, case series, studies that did not examine the prevalence of toxoplasmosis, and studies without sheep and goats were excluded. A PRISMA flowchart of the selection of articles is presented in Figure 6.

A total of 17 articles were chosen based on the study’s inclusion criteria. The following information about the included articles were collected: year of publication, country where the study was conducted, sample size, diagnostic tests, number of animals tested, number of cases of infected animals, and prevalence rates. The studies were then coded as per the defined parameters, and the data were entered into Microsoft Excel.

### 4.4. Meta-Analysis

The weighted prevalence of *T. gondii* infection was used in the analyses of sheep and goats by sex (i.e., male and female) and age group, i.e., group 1 (≤1 year of age) and group 2 (>1 year of age). We used ORs for the pooled effect sizes of both analyses; the Sidik–Jonkman estimator was used to estimate the variance, and 0.1 increments were added for a continuity correction of zero cells. However, for the proportions of the infected cases by sex and age group, we analyzed single proportions. We used the Freeman–Tukey double arcsine transformation for the analyses of the proportions, and the DerSimonian–Laird method to estimate the inverse variances. Cochran’s Q test and the $I^{2}$ Statistics were used to determine heterogeneity between the studies, and forest plots with 95% Cls and effect sizes were used to present a graphical summary of the results.

## 5. Conclusions

Examining these findings contributes to an updated epidemiological assessment and geographic context in Pakistan. More surveys are recommended to monitor this infection continuously. Attention should be paid to farming and testing animals using techniques to control the disease before a contaminated product is obtained and consumed. Health measures and precautions should be taken to prevent and control the disease. Our data provide meaningful information and statistics on the prevalence of toxoplasmosis, which may aid in the disease’s control and management. Additional investigations are needed to improve control strategies, reduce toxoplasmosis among goats and sheep, and continuously buffer Pakistan’s community health, economy, and financial status against the societal damages caused by toxoplasmosis.

## Authors Contributions

Conceptualization and Design, T.M (Tanzila Mumtaz)., A.A. and U.A.A.; Analysis and Interpretation of Data, T.M (Tahir Mahmood) and A.M. (Aqsa Mushtaq); Writing—Original Draft Preparation, T.M (Tahir Mahmood)., U.A.A., K.A., S.W. and T.M (Teroj Mohamed).; Statistical Analysis, T.M. (Tahir Mahmood) and T.M. (Tanzila Mohamed); Supervision, H.A., M.S.A. and J.C.; Writing—Review and Editing, H.A., A.M. (Ali Muhammad), U.A.A., M.S.A., H.L. and J.C. All authors have read and agreed to the published version of the manuscript.

## Figures and Tables

**Table 1:** Year(s) the study was conducted and prevalence of *T. gondii* infection among sheep and goats by province.

No.	Year(s) the Study Was Conducted	Reference	Province(s)	Animal						Lab Test
				Sheep			Goats			
				Total	Positive	%	Total	Positive	%	
1	2006–2007	Ramzan et al. [57].	Southern Punjab	90	10	11.11	110	28	25.45	LAT
2	2010	Lashari and Tasawar [58]	Southern Punjab	518	103	19.88				LAT ELISA
3	2011	Tasawar et al. [54]	Southern Punjab				200	104	52	LAT
4	2011–2012	Ahmad et al. [59]	Northern Punjab	413	75	18.15	419	60	14.31	ELISA
5	2012–2013	Hanif and Tasawar [60]	Southern Punjab	288	81	28.12				ELISA
			Southern Punjab	212	56	26.41				ELISA
			Southern Punjab	288	98	34.03				LAT
			Southern Punjab	212	70	33.02				LAT
										
6	2013	Shah et al. [61]	Khyber Pakhtunkhwa	350	148	42.28	290	128	44.12	IHA
7	2013	Ahmed et al. [62]	Central Punjab	113	18	15.92	86	38	44.18	ELISA
			Central Punjab	70	23	32.86	105	46	43.8	ELISA
			Central Punjab	90	7	7.77	74	19	25.67	ELISA
			Central Punjab	137	46	33.57	148	53	35.81	ELISA
			Central Punjab	60	29	48.33	117	71	60.68	ELISA
8	2013	Shah et al. [61]	Khyber Pakhtunkhwa	100	36	36	104	56	53.84	IHA
9	2012–2013	Ahmad and Tasawar [55]	Southern Punjab	335	125	37.31				LAT
10	2015	Ullah et al. [47]	Southern Punjab	55	20	36.36	55	21	31.18	LAT
			Southern Punjab	40	18	45	40	12	30	LAT
			Southern Punjab	30	18	60	30	18	60	LAT
11	2016	Ahmad and Tasawar [63]	Southern Punjab	335	125	37.3	865	252	29.13	LAT
12	2018	Khan et al. [64]	Khyber Pakhtunkhwa				65	30	46.15	LAT
			Khyber Pakhtunkhwa				61	20	32.79	LAT
			Khyber Pakhtunkhwa				23	12	52.17	LAT
13	2018	Khan et al. [64]	Khyber Pakhtunkhwa				68	1	1.47	ELISA
14	2019	Ahmed et al. [65]	Northern Punjab	45	15	33.33	46	11	23.91	ELISA
15	2019	Hussain and Zahid [66]	Khyber Pakhtunkhwa	103	89	86.4	121	99	81.82	LAT
16	2019	Kamal et al. [67]	Khyber Pakhtunkhwa	66	25	37.88				LAT
			Khyber Pakhtunkhwa	42	19	45.24				LAT
			Khyber Pakhtunkhwa	35	14	40				LAT
17	2020	Lashari et al. [68]	Southern Punjab	103	24	23.3	101	33	32.6	ELISA
			Southern Punjab	103	26	25.24	101	36	35.64	LAT

LAT, latex agglutination test; ELISA, enzyme-linked immunosorbent assay; IHA, indirect hemagglutination assay.

**Table 2:** Year of publication and prevalence of *T. gondii* infection among sheep and goats by province and city.

Year of Publication	Reference	Province(s)	City	Animal						Lab Method
				Sheep			Goats			
				Total	Positive	%	Total	Positive	%	
2009	Ramzan et al. [57]	Southern Punjab	Rahim Yar Khan	90	10	11.11	110	28	25.45	LAT
2010	Lashari and Tasawar [58]	Southern Punjab	Dera Ghazi Khan, Multan and Khanewal	518	103	19.88				LAT ELISA
2011	Tasawar et al. [54]	Southern Punjab	Multan				200	104	52	LAT
2015	Ahmad et al. [59]	Northern Punjab	Pothwar region	413	75	18.15	419	60	14.31	ELISA
2016	Hanif and Tasawar [60]	Southern Punjab	Multan	288	98	34.03				LAT
		Southern Punjab	Khanewal	212	70	33.02				LAT
		Southern Punjab	Multan	288	81	28.12				ELISA
		Southern Punjab	Khanewal	212	56	26.41				ELISA
2013	Shah et al. [61]	KPK	Mardan	350	148	42.28	290	128	44.12	IHA
2016	Ahmed et al. [62]	Central Punjab	Bhalwal	113	18	15.92	86	38	44.18	ELISA
		Central Punjab	Sahiwal	70	23	32.86	105	46	43.8	ELISA
		Central Punjab	Shahpur	90	7	7.77	74	19	25.67	ELISA
		Central Punjab	Silanwali	137	46	33.57	148	53	35.81	ELISA
		Central Punjab	Sargodha	60	29	48.33	117	71	60.68	ELISA
2013	Shah et al. [61]	KPK	Mohmand Agency (Khazeena, Nawagai, Chamarkan, Ulai And Ghalana)	100	36	36	104	56	53.84	IHA
2015	Ahmad and Tasawar [55]	Southern Punjab	Cholistan	335	125	37.31				LAT
2018	Ullah et al. [47]	Southern Punjab	Multan	55	20	36.36	55	21	31.18	LAT
		Southern Punjab	Shujabad	40	18	45	40	12	30	LAT
		Southern Punjab	Jalalpur	30	18	60	30	18	60	LAT
2016	Ahmad and Tasawar [63]	Southern Punjab	Cholistan Desert, Rahim Yar Khan And Rajan Pur	335	125	37.31	865	252	29.13	LAT
2018	Khan et al. [64]	KPK	Charsada				65	30	46.15	LAT
		KPK	Tangi				61	20	32.79	LAT
		KPK	Shabqadar				23	12	52.17	LAT
2018	Khan et al. [64]	KPK	District Bannu				68	1	1.47	LAT
2019	Ahmed et al. [65]	Potohar (northern punjab)	Jhelum, Chakwal, Rawalpindi, Attock, Islamabad	45	15	33.33	46	11	23.91	LAT ELISA
2019	Hussain and Zahid [66]	KPK	Charsada (Shabqadar, Tangi and Charsada)	103	89	86.4	121	99	81.82	LAT
2019	Kamal et al. [67]	KPK	Charsada	66	25	37.88				
		KPK	Tangi	42	19	45.24				
								
		KPK	Shabqadar	35	14	40				
2020	Lashari et al. [68]	Southern Punjab	DG Khan	103	26	25.24	101	36	35.64	LAT
		Southern Punjab	DG Khan	103	24	23.3	101	33	32.6	LAT

LAT, latex agglutination test; ELISA, enzyme-linked immunosorbent assay; IHA, indirect hemagglutination assay.

**Table 3:** Prevalence of *T. gondii* infection among sheep and goats by sex.

Reference	Province	City	Sheep							Goats						
			Total	Male	NI	%	Female	NI	%	Total	Male	NI	%	Female	NI	%
Ahmad et al. [59]	Northern Punjab	Pothwar Region	413	156	20	12.82	257	55	21.4	419	153	16	10.46	266	44	16.54
Lashari and Tasawar [58]	Southern Punjab	Dera Ghazi Khan, Multan and Khanewal	518	63	19	30.15	455	84	18.4							
Ramzan et al. [57]	Southern Punjab	Rahim Yar Khan	90	44	2	4.5	46	8	17.3	110	62	10	16.1	48	18	37.5
Ahmed et al. [62]	Central Punjab	Bhalwal, Kotmomin, Sahiwal, Shahpur, Silanwali, and Sargodha	470	72	16	22.2	398	107	26.9	530	150	35	23.3	380	192	50.5
Shah et al. [61]	Khyber Pakhtunkhwa	Mardan	290	120	55	45.83	170	73	42.94	350	150	39	26	200	109	54.5
Tasawar et al. [54]	Southern Punjab	Multan								200	20	5	25	180	99	55
Hanif and Tasawar [60]	Southern Punjab	Multan, Khanewal	500 LAT	51	16	31.37	449	152	33.85							
			500 ELISA	51	10	19.6	449	127	28.28							
Ullah et al. [47]	Southern Punjab	Multan	125	63	16	25.39	62	40	64.52	125	63	15	23.81	62	36	58.06
Khan et al. [64]	Khyber Pakhtunkhwa	Charsadda								149	56	18	32.14	93	44	47.31
Shah et al. [61]	Khyber Pakhtunkhwa	Mohmand agency	100	52	16	30.76	48	20	41.6	104	52	20	38.46	52	36	69.23
Lashari et al. [68]	Southern Punjab	D.G. Khan district	103 LAT	15	5	33.3	88	21	23.86	101 LAT	8	3	37.5	93	33	35.48
			103 ELISA	15	5	33.3	88	19	21.59	101 ELISA	8	2	25	93	31	33.3
Hussain and Zahid [66]	Khyber Pakhtunkhwa	Charsadda	103	33	25	84.78	70	64	91.42	121	29	21	72.4	92	78	84.78
Kamal et al. [67]	Khyber Pakhtunkhwa	Charsadda	143	78	26	33.3	65	32	49.23							
Ahmad and Tasawar [55]	Southern Punjab	Cholistan	335	169	52	30.7	166	73	43.9							

NI, number of animals infected; LAT, latex agglutination test; ELISA, enzyme-linked immunosorbent assay; IHA, indirect hemagglutination assay.

**Table 4:** Prevalence of *T. gondii* infection among sheep and goats by age group.

Reference	Province	Sheep						Goats					
		Total Positives	%	Age Group	No. in Age Group	No. of Positives in Age Group	Percentage of Positives in Age Group	Total Positives	%	Age Group	No. in Age Group	No. of Positives in Age Group	Percentage of Positives in Age Group
Ahmad et al. [59]	Northern Punjab	75	18.16	<12	44	4	9.09	60	14.31	<12	30	3	10.0
				13–24	174	16	9.20			13–24	181	15	8.29
				25–36	138	35	25.36			25–36	137	23	16.79
				>36	57	20	35.09			>36	71	19	26.76
Lashari and Tasawar [58]	Southern Punjab	103	19.88	3–15 months	88	12	13.6						
				16–28 months	54	21	38.8						
				29–41 months	137	33	24.08						
				42–54 months	117	21	17.94						
				55–67 months	75	12	16.0						
				68–80 months	47	4	8.51						
Ramzan et al. [57]	Southern Punjab	10	11.11	≤1 year	14	0	0	28	25.45	≤1 year	23	6	26
				1–1.5 years	35	2	5.7			1–1.5 years	44	18	40.9
				2–2.5 years	32	6	18.7			2–2.5 years	26	4	15.3
				≥3 years	9	2	22.7			≥3 years	17	0	0
Ahmed et al. [62]	Central Punjab	227	42.8	≤1.5 year	193	71	36.8	123	26.2	≤1.5 year	206	27	13.1
				1.5–3 year	122	49	40.2			1.5–3 year	105	33	31.4
				≥3 year	215	107	49.8			≥3 year	159	63	39.6
Shah et al. [61]	Khyber Pakhtunkhwa	128	44.12	≤1 year	60	8	13.33	148	42.28	≤1 year	50	10	20
				1–2 year	110	40	36.36			1–2 year	120	40	33.33
				≥2 year	120	80	66.66			≥2 year	180	98	54.44
Hanif and Tasawar [60]	Southern Punjab	168 (LAT)	33.6	4–7 months	125	33	26.40						
				18–31 months	152	49	32.33						
				32–45 months	120	37	30.83						
				46–59 months	72	29	40.27						
				60–73 months	31	20	64.50						
		137 (ELISA)	27.4	4–7 months	125	28	22.40						
				18–31 months	152	43	28.28						
				32–45 months	120	32	26.66						
				46–59 months	72	22	30.55						
				60–73 months	31	12	38.70						
Ullah et al. [47]	Southern Punjab	56	44.8	Less than 1 year	11	03	27.27	51	40.8	Less than 1 year	09	01	11.1
				>1–1.5 year	50	30	60			>1–1.5 year	45	25	55.56
				>1.5–2 year	59	22	37.28			>1.5–2 year	59	23	38.99
				>2 year	05	01	20			>2 year	12	02	16.67
Ahmad and Tasawar [63]	Southern Punjab (goat and sheep combined data)			1–6 months	374	64	17.11						
				7–12 months	338	75	22.18						
				13–18 months	289	127	43.94						
				19–24 months	114	65	57.01						
				>25 months	85	46	54.11						
Khan et al. [64]	Khyber Pakhtunkhwa							62	41.61	≤1 year	33	10	30.30
										1–2 year	33	9	25.71
										2–3 year	42	24	57.14
										>3 year	39	19	48.71
Lashari et al. [68]	Southern Punjab	26 (LAT)	25.24	8–9	34	7	20.58	26 (LAT)	25.71	8–21	29	5	17.24
				20–31	42	7	16.6			22–35	42	14	33.33
				32–42	27	12	44.4			36–49	30	7	56.66
		24 (ELISA)	23.3	8–9	34	5	14.70	23 (ELISA)	22.77	8–21	29	4	13.79
				20–31	31	9	21.95			22–35	42	12	28.57
				32–42	28	10	35.71			36–49	30	7	56.66
Kamal et al. [67]	Khyber Pakhtunkhwa	48	33.56	≤1 year	27	9	33.33						
				1–2 year	43	17	39.53						
				2–3 year	45	19	42.22						
				>3year	28	13	46.42						

LAT, latex agglutination test; ELISA, enzyme-linked immunosorbent assay.

**Table 7:** Overall prevalence of *T. gondii* infection in sheep by age group.

Author(s) *	Age ≤ 1 Year	Age > 1 Year	OR	95% CI	%W (Fixed)	%W (Random)
	Infected/Total	Infected/Total				
Ahmad et al. [59]	4/44	71/369	0.4197	[0.1454; 1.2113]	3.8	11.2
Ramzan et al. [57]	0/14	10/76	0.0464	[0.0001; 24.1651]	0.9	0.6
Shah et al. [61]	8/60	120/230	0.141	[0.0641; 0.3101]	11.9	14.6
Hanif and Tasawar [60]	28/125	109/375	0.7044	[0.4377; 1.1338]	11.7	19.2
Ullah et al. [47]	3/11	53/114	0.4316	[0.1089; 1.7104]	1.9	8.2
Ahmad and Tasawar [55]	139/712	238/488	0.2548	[0.1971; 0.3294]	63.1	22
Lashari et al. [68]	5/34	19/59	0.3630	[0.1214; 1.0850]	3.3	10.8
Kamal et al. [67]	9/27	49/116	0.6837	[0.2833; 1.6497]	3.4	13.4
					z|t	*p*-value
Total (fixed effects)	196/1027	669/1827	0.3200	[0.2617; 0.3913]	−11.11	<0.0001
Total (random effects)	196/1027	669/1827	0.3681	[0.2210; 0.6132]	−4.63	0.0024

* Out of 10 articles, 2 articles were not included due to the variation in the age group data. OR, odds ratio; CI, confidence interval.

**Table 8:** Overall prevalence of *T. gondii* infection among goats by age group.

Author(s) *	Age ≤ 1 Year	Age > 1 Year	OR	95% CI	%W (Fixed)	%W (Random)
	Infected/Total	Infected/Total				
Ahmad et al. [59]	3/30	57/389	0.6472	[0.1900; 2.2041]	9.4	13.9
Ramzan et al. [57]	6/23	22/87	1.0428	[0.3654; 2.9762]	8.7	17.2
Shah et al. [61]	10/50	138/300	0.2935	[0.1415; 0.6085]	40.6	25.7
Ullah et al. [47]	1/9	50/116	0.165	[0.0200; 1.3624]	8.2	5.8
Khan et al. [64]	10/33	52/114	0.5184	[0.2263; 1.1875]	20.9	22.6
Lashari et al. [68]	4/29	19/72	0.4463	[0.1374; 1.4502]	12.1	14.7
					z|t	*p*-value
Total (fixed effects)	34/174	338/1078	0.4474	[0.2972; 0.6734]	−3.86	0.0001
Total (random effects)	34/174	338/1078	0.4768	[0.2964; 0.8438]	−3.34	0.0207

* Two of the seven articles were not included due to the variation in the age group data. OR, odds ratio; CI, confidence interval.

## Data Availability

Not applicable.
